# *catena*-Poly[[(acetato-κ^2^ *O*,*O*′)(methanol-κ*O*)cadmium(II)]-μ-[1,2-bis­(1*H*-benzimid­azol-2-yl)ethane]-κ^2^ *N* ^3^:*N* ^3′^-[(acetato-κ^2^ *O*,*O*′)(methanol-κ*O*)cadmium(II)]-di-μ-chlorido]

**DOI:** 10.1107/S1600536810014753

**Published:** 2010-04-28

**Authors:** Huai-Xia Yang, Jun Zhang, Ya-Nan Ding, Xiang-Ru Meng

**Affiliations:** a Pharmacy College, Henan University of Traditional Chinese Medicine, Zhengzhou 450008, People’s Republic of China; b Department of Chemistry, Zhengzhou University, Zhengzhou 450052, People’s Republic of China

## Abstract

In the title complex, [Cd_2_(CH_3_COO)_2_Cl_2_(C_16_H_14_N_4_)(CH_3_OH)_2_]_*n*_, the Cd^II^ atom is six-coordinated by one N atom from a centrosymmetric bridging 1,2-bis­(2,2′-1*H*-benzimidazol-2-yl)ethane (bbe) ligand, two O atoms from a chelating acetate ligand, one O atom from a methanol mol­ecule and two bridging Cl atoms in a distorted octa­hedral geometry. The Cd^II^ atoms are connected alternately by the Cl atoms and bbe ligands, leading to a chain along [001]. These chains are further linked by O—H⋯O hydrogen bonds. Intra­chain N—H⋯O hydrogen bonds are observed.

## Related literature

For metal complexes of 1,2-bis­(2,2′-1*H*-benzimidazole)ethane, see: van Albada *et al.* (2007 ▶); Shen & Yuan (2006 ▶). For related Cd(II) complexes, see: Yam & Lo (1999 ▶); Zhai *et al.* (2006 ▶).
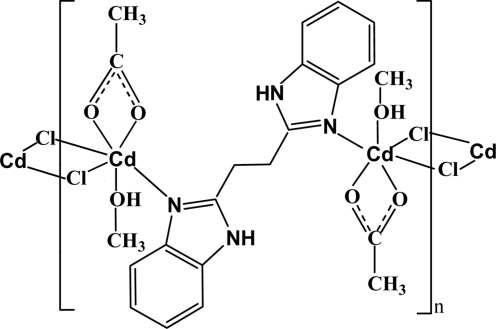


## Experimental

### 

#### Crystal data

[Cd_2_(C_2_H_3_O_2_)_2_Cl_2_(C_16_H_14_N_4_)(CH_4_O)_2_]
*M* *_r_* = 740.20
Triclinic, 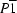

*a* = 7.3983 (15) Å
*b* = 9.6391 (19) Å
*c* = 10.228 (2) Å
α = 96.79 (3)°
β = 98.15 (3)°
γ = 90.45 (3)°
*V* = 716.7 (3) Å^3^
*Z* = 1
Mo *K*α radiation
μ = 1.71 mm^−1^
*T* = 293 K
0.18 × 0.16 × 0.13 mm

#### Data collection

Rigaku Saturn CCD diffractometer
Absorption correction: multi-scan (*CrystalClear*; Rigaku/MSC, 2006 ▶) *T*_min_ = 0.748, *T*_max_ = 0.808
8831 measured reflections
3396 independent reflections
3143 reflections with *I* > 2σ(*I*)
*R*_int_ = 0.024

#### Refinement

*R*[*F* ^2^ > 2σ(*F* ^2^)] = 0.027
*wR*(*F* ^2^) = 0.057
*S* = 1.05
3396 reflections
163 parameters
H-atom parameters constrained
Δρ_max_ = 0.51 e Å^−3^
Δρ_min_ = −0.37 e Å^−3^

### 

Data collection: *CrystalClear* (Rigaku/MSC, 2006 ▶); cell refinement: *CrystalClear*; data reduction: *CrystalClear*; program(s) used to solve structure: *SHELXS97* (Sheldrick, 2008 ▶); program(s) used to refine structure: *SHELXL97* (Sheldrick, 2008 ▶); molecular graphics: *SHELXTL* (Sheldrick, 2008 ▶); software used to prepare material for publication: *SHELXTL*.

## Supplementary Material

Crystal structure: contains datablocks global, I. DOI: 10.1107/S1600536810014753/hy2299sup1.cif

Structure factors: contains datablocks I. DOI: 10.1107/S1600536810014753/hy2299Isup2.hkl

Additional supplementary materials:  crystallographic information; 3D view; checkCIF report

## Figures and Tables

**Table 1:** Hydrogen-bond geometry (Å, °)

*D*—H⋯*A*	*D*—H	H⋯*A*	*D*⋯*A*	*D*—H⋯*A*
N2—H2*A*⋯O2^i^	0.86	2.04	2.791 (3)	145
O3—H3*B*⋯O1^ii^	0.82	1.83	2.646 (3)	175

Symmetry codes: (i) 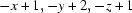; (ii) 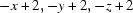.

**Fig. 1.**
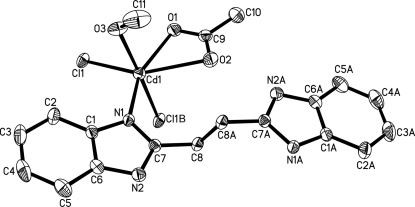
The asymmetric unit of the title complex. Displacement ellipsoids are drawn at the 30% probability level. H atoms have been omitted for clarity. [Symmetry codes: (A) -x+1, -y+2, -z+1; (B) -x+1, -y+2, -z+2.]

**Fig. 2.**
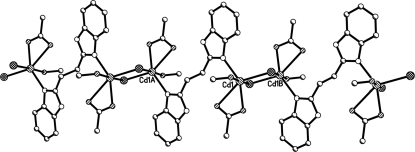
View of the one-dimensional chain in the title complex. [Symmetry codes: (A) -x+1, -y+2, -z+1; (B) -x+1, -y+2, -z+2.]

## supplementary crystallographic information

### Comment

1,2-Bis(2,2'-1*H*-benzimidazole)ethane (bbe) as a multidentate ligand has been extensively used in the construction of metal complexes due to strong coordination ability of the N-donor (van Albada *et al.*, 2007; Shen & Yuan, 2006). In addition, Cd^II^ ion is a favorable and fashionable building block or connecting node, not only for it is easy to coordinate to N/O-containing ligands, but also the closed-shell d^10^ Cd–Cd interaction can often give rise to intriguing supramolecular motifs and properties (Yam & Lo, 1999; Zhai *et al.*, 2006). In this work, through the self-assembly of bbe hydrochloride with cadmium acetate at room temperature, we obtained the title complex.

In the title complex, the Cd^II^ ion is six-coordinated by three O atoms from a chelating acetate ligand and a methanol molecule, one N atom from a bbe ligand and two Cl atoms, leading to a distorted octahedral geometry (Fig. 1). The two Cd^II^ ions are connected by a pair of bridging Cl atoms, yielding a Cd_2_Cl_2_ binuclear unit with a Cd···Cd distance of 3.667 (1) Å. The dimers are further linked by bbe ligands to give a one-dimensional chain along [0 0 1] (Fig. 2). The distance between two Cd atoms bridged by the bbe ligand is 7.722 (2) Å. In addition, there are N—H···O hydrogen bonds between bbe and acetate group, and O—H···O hydrogen bonds between methanol molecule and acetate group (Table 1). The linear chains are linked through O—H···O hydrogen bonds.

### Experimental

1,2-Bis(2,2'-1*H*-benzimidazole)ethane hydrochloride (0.05 mmol) in methanol (6 ml) was added dropwise to an aqueous solution (2 ml) of cadmium acetate (0.05 mmol). The resulting solution was allowed to stand at room temperature. After one week colorless crystals with good quality were obtained from the filtrate and dried in air.

### Refinement

H atoms were positioned geometrically and refined as riding atoms, with C—H = 0.93 (aromatic), 0.97 (CH_2_) and 0.96 (CH_3_) Å and O—H = 0.82 Å, and with *U*_iso_(H) = 1.2(1.5 for methyl)*U*_eq_(C, O).

### Crystal data

**Table:**

[Cd_2_(C_2_H_3_O_2_)_2_Cl_2_(C_16_H_14_N_4_)(CH_4_O)_2_]	*Z* = 1
*M**_r_* = 740.20	*F*(000) = 366
Triclinic, *P*1	*D*_x_ = 1.715 Mg m^−^^3^
Hall symbol: -P 1	Mo *K*α radiation, λ = 0.71073 Å
*a* = 7.3983 (15) Å	Cell parameters from 2503 reflections
*b* = 9.6391 (19) Å	θ = 2.0–27.9°
*c* = 10.228 (2) Å	µ = 1.71 mm^−^^1^
α = 96.79 (3)°	*T* = 293 K
β = 98.15 (3)°	Prism, colorless
γ = 90.45 (3)°	0.18 × 0.16 × 0.13 mm
*V* = 716.7 (3) Å^3^	

### Data collection

**Table:**

Rigaku Saturn CCD diffractometer	3396 independent reflections
Radiation source: fine-focus sealed tube	3143 reflections with *I* > 2σ(*I*)
graphite	*R*_int_ = 0.024
Detector resolution: 28.5714 pixels mm^-1^	θ_max_ = 27.9°, θ_min_ = 3.1°
ω scans	*h* = −9→9
Absorption correction: multi-scan (*CrystalClear*; Rigaku/MSC, 2006)	*k* = −12→11
*T*_min_ = 0.748, *T*_max_ = 0.808	*l* = −13→13
8831 measured reflections	

### Refinement

**Table:**

Refinement on *F*^2^	Primary atom site location: structure-invariant direct methods
Least-squares matrix: full	Secondary atom site location: difference Fourier map
*R*[*F*^2^ > 2σ(*F*^2^)] = 0.027	Hydrogen site location: inferred from neighbouring sites
*wR*(*F*^2^) = 0.057	H-atom parameters constrained
*S* = 1.05	*w* = 1/[σ^2^(*F*_o_^2^) + (0.0236*P*)^2^ + 0.3424*P*] where *P* = (*F*_o_^2^ + 2*F*_c_^2^)/3
3396 reflections	(Δ/σ)_max_ = 0.001
163 parameters	Δρ_max_ = 0.51 e Å^−^^3^
0 restraints	Δρ_min_ = −0.37 e Å^−^^3^

### Fractional atomic coordinates and isotropic or equivalent isotropic displacement parameters (Å^2^)

**Table:**

	*x*	*y*	*z*	*U*_iso_*/*U*_eq_	
Cd1	0.64998 (2)	0.967293 (18)	0.872095 (16)	0.03484 (6)	
Cl1	0.63872 (9)	0.88042 (7)	1.09623 (6)	0.04316 (14)	
N1	0.4927 (3)	0.82344 (19)	0.70445 (18)	0.0317 (4)	
N2	0.3205 (3)	0.7493 (2)	0.51282 (19)	0.0371 (4)	
H2A	0.2596	0.7510	0.4349	0.045*	
O1	0.8449 (2)	1.1502 (2)	0.94761 (18)	0.0490 (4)	
O2	0.7646 (3)	1.1554 (2)	0.73532 (18)	0.0539 (5)	
O3	0.8976 (2)	0.8247 (2)	0.84264 (18)	0.0534 (5)	
H3B	0.9727	0.8331	0.9104	0.064*	
C1	0.4503 (3)	0.6812 (2)	0.7024 (2)	0.0334 (5)	
C2	0.4968 (4)	0.5910 (3)	0.7977 (3)	0.0507 (7)	
H2B	0.5683	0.6214	0.8785	0.061*	
C3	0.4331 (5)	0.4555 (3)	0.7678 (4)	0.0632 (9)	
H3A	0.4624	0.3927	0.8295	0.076*	
C4	0.3255 (5)	0.4095 (3)	0.6473 (4)	0.0623 (9)	
H4A	0.2849	0.3167	0.6308	0.075*	
C5	0.2775 (4)	0.4969 (3)	0.5522 (3)	0.0527 (7)	
H5A	0.2052	0.4660	0.4718	0.063*	
C6	0.3430 (3)	0.6343 (2)	0.5826 (2)	0.0368 (5)	
C7	0.4119 (3)	0.8581 (2)	0.5898 (2)	0.0309 (4)	
C8	0.4232 (3)	0.9973 (2)	0.5432 (2)	0.0341 (5)	
H8A	0.4464	1.0686	0.6192	0.041*	
H8B	0.3076	1.0166	0.4917	0.041*	
C9	0.8471 (3)	1.2083 (3)	0.8436 (3)	0.0405 (5)	
C10	0.9492 (5)	1.3457 (3)	0.8554 (4)	0.0669 (9)	
H10A	0.9396	1.3780	0.7695	0.100*	
H10B	1.0754	1.3337	0.8886	0.100*	
H10C	0.8977	1.4130	0.9156	0.100*	
C11	0.9830 (5)	0.8087 (5)	0.7288 (3)	0.0873 (13)	
H11A	1.0811	0.7447	0.7404	0.131*	
H11B	1.0306	0.8976	0.7142	0.131*	
H11C	0.8960	0.7727	0.6534	0.131*	

### Atomic displacement parameters (Å^2^)

**Table:**

	*U*^11^	*U*^22^	*U*^33^	*U*^12^	*U*^13^	*U*^23^
Cd1	0.03432 (10)	0.04266 (11)	0.02727 (9)	−0.00239 (7)	0.00436 (7)	0.00344 (7)
Cl1	0.0498 (3)	0.0495 (3)	0.0348 (3)	0.0145 (3)	0.0123 (3)	0.0158 (3)
N1	0.0350 (10)	0.0313 (9)	0.0289 (9)	−0.0008 (8)	0.0022 (8)	0.0070 (7)
N2	0.0423 (11)	0.0367 (10)	0.0302 (10)	−0.0059 (9)	0.0005 (8)	0.0012 (8)
O1	0.0458 (10)	0.0620 (12)	0.0364 (9)	−0.0118 (9)	−0.0059 (8)	0.0099 (8)
O2	0.0629 (12)	0.0590 (12)	0.0352 (10)	−0.0100 (10)	−0.0075 (9)	0.0055 (8)
O3	0.0368 (10)	0.0817 (14)	0.0369 (10)	0.0127 (9)	−0.0015 (8)	−0.0047 (9)
C1	0.0332 (11)	0.0311 (11)	0.0380 (12)	0.0013 (9)	0.0096 (10)	0.0074 (9)
C2	0.0568 (17)	0.0436 (14)	0.0555 (17)	0.0045 (12)	0.0074 (13)	0.0224 (13)
C3	0.073 (2)	0.0418 (15)	0.084 (2)	0.0060 (15)	0.0231 (18)	0.0302 (16)
C4	0.073 (2)	0.0295 (13)	0.091 (3)	−0.0046 (13)	0.0337 (19)	0.0073 (15)
C5	0.0550 (17)	0.0408 (14)	0.0613 (18)	−0.0101 (12)	0.0169 (14)	−0.0068 (13)
C6	0.0372 (12)	0.0336 (12)	0.0405 (13)	−0.0045 (10)	0.0106 (10)	0.0018 (10)
C7	0.0330 (11)	0.0317 (11)	0.0283 (10)	−0.0010 (9)	0.0056 (9)	0.0036 (8)
C8	0.0411 (13)	0.0331 (11)	0.0285 (11)	0.0027 (10)	0.0026 (10)	0.0079 (9)
C9	0.0353 (12)	0.0451 (14)	0.0392 (13)	−0.0015 (10)	−0.0001 (10)	0.0041 (11)
C10	0.063 (2)	0.0548 (18)	0.079 (2)	−0.0157 (15)	−0.0042 (17)	0.0111 (16)
C11	0.059 (2)	0.145 (4)	0.053 (2)	0.023 (2)	0.0150 (17)	−0.017 (2)

### Geometric parameters (Å, °)

**Table:**

Cd1—N1	2.250 (2)	C2—H2B	0.9300
Cd1—O1	2.260 (2)	C3—C4	1.391 (5)
Cd1—O3	2.3307 (19)	C3—H3A	0.9300
Cd1—Cl1	2.5438 (8)	C4—C5	1.373 (4)
Cd1—O2	2.622 (2)	C4—H4A	0.9300
Cd1—Cl1^i^	2.6372 (10)	C5—C6	1.391 (3)
Cl1—Cd1^i^	2.6372 (10)	C5—H5A	0.9300
N1—C7	1.319 (3)	C7—C8	1.482 (3)
N1—C1	1.401 (3)	C8—C8^ii^	1.539 (4)
N2—C7	1.349 (3)	C8—H8A	0.9700
N2—C6	1.387 (3)	C8—H8B	0.9700
N2—H2A	0.8600	C9—C10	1.503 (4)
O1—C9	1.262 (3)	C10—H10A	0.9600
O2—C9	1.236 (3)	C10—H10B	0.9600
O3—C11	1.395 (4)	C10—H10C	0.9600
O3—H3B	0.8200	C11—H11A	0.9600
C1—C6	1.387 (3)	C11—H11B	0.9600
C1—C2	1.391 (3)	C11—H11C	0.9600
C2—C3	1.370 (4)		
			
N1—Cd1—O1	151.13 (7)	C4—C3—H3A	119.2
N1—Cd1—O3	86.00 (7)	C5—C4—C3	122.0 (3)
O1—Cd1—O3	89.72 (7)	C5—C4—H4A	119.0
N1—Cd1—Cl1	111.71 (5)	C3—C4—H4A	119.0
O1—Cd1—Cl1	96.76 (5)	C4—C5—C6	116.2 (3)
O3—Cd1—Cl1	89.40 (6)	C4—C5—H5A	121.9
N1—Cd1—O2	99.39 (6)	C6—C5—H5A	121.9
O1—Cd1—O2	52.27 (6)	C1—C6—N2	105.83 (19)
O3—Cd1—O2	92.78 (7)	C1—C6—C5	122.2 (2)
Cl1—Cd1—O2	148.90 (5)	N2—C6—C5	132.0 (2)
N1—Cd1—Cl1^i^	92.23 (6)	N1—C7—N2	112.48 (19)
O1—Cd1—Cl1^i^	92.53 (6)	N1—C7—C8	125.8 (2)
O3—Cd1—Cl1^i^	177.71 (5)	N2—C7—C8	121.7 (2)
Cl1—Cd1—Cl1^i^	89.91 (3)	C7—C8—C8^ii^	110.6 (2)
O2—Cd1—Cl1^i^	88.94 (5)	C7—C8—H8A	109.5
Cd1—Cl1—Cd1^i^	90.09 (3)	C8^ii^—C8—H8A	109.5
C7—N1—C1	105.53 (18)	C7—C8—H8B	109.5
C7—N1—Cd1	126.73 (15)	C8^ii^—C8—H8B	109.5
C1—N1—Cd1	127.64 (15)	H8A—C8—H8B	108.1
C7—N2—C6	107.29 (19)	O2—C9—O1	121.1 (2)
C7—N2—H2A	126.4	O2—C9—C10	120.8 (3)
C6—N2—H2A	126.4	O1—C9—C10	118.1 (2)
C9—O1—Cd1	101.50 (15)	C9—C10—H10A	109.5
C9—O2—Cd1	84.98 (16)	C9—C10—H10B	109.5
C11—O3—Cd1	125.0 (2)	H10A—C10—H10B	109.5
C11—O3—H3B	111.3	C9—C10—H10C	109.5
Cd1—O3—H3B	110.3	H10A—C10—H10C	109.5
C6—C1—C2	120.6 (2)	H10B—C10—H10C	109.5
C6—C1—N1	108.9 (2)	O3—C11—H11A	109.5
C2—C1—N1	130.5 (2)	O3—C11—H11B	109.5
C3—C2—C1	117.3 (3)	H11A—C11—H11B	109.5
C3—C2—H2B	121.3	O3—C11—H11C	109.5
C1—C2—H2B	121.3	H11A—C11—H11C	109.5
C2—C3—C4	121.6 (3)	H11B—C11—H11C	109.5
C2—C3—H3A	119.2		
			
N1—Cd1—Cl1—Cd1^i^	−92.37 (6)	C7—N1—C1—C6	0.3 (3)
O1—Cd1—Cl1—Cd1^i^	92.53 (6)	Cd1—N1—C1—C6	176.73 (15)
O3—Cd1—Cl1—Cd1^i^	−177.82 (5)	C7—N1—C1—C2	−178.9 (3)
O2—Cd1—Cl1—Cd1^i^	87.79 (10)	Cd1—N1—C1—C2	−2.5 (4)
Cl1^i^—Cd1—Cl1—Cd1^i^	0.0	C6—C1—C2—C3	0.2 (4)
O1—Cd1—N1—C7	−38.2 (3)	N1—C1—C2—C3	179.4 (3)
O3—Cd1—N1—C7	−120.30 (19)	C1—C2—C3—C4	−0.2 (5)
Cl1—Cd1—N1—C7	151.94 (17)	C2—C3—C4—C5	0.0 (5)
O2—Cd1—N1—C7	−28.1 (2)	C3—C4—C5—C6	0.2 (4)
Cl1^i^—Cd1—N1—C7	61.15 (19)	C2—C1—C6—N2	179.1 (2)
O1—Cd1—N1—C1	146.09 (18)	N1—C1—C6—N2	−0.2 (3)
O3—Cd1—N1—C1	63.97 (19)	C2—C1—C6—C5	0.0 (4)
Cl1—Cd1—N1—C1	−23.78 (19)	N1—C1—C6—C5	−179.3 (2)
O2—Cd1—N1—C1	156.13 (18)	C7—N2—C6—C1	0.1 (3)
Cl1^i^—Cd1—N1—C1	−114.58 (18)	C7—N2—C6—C5	179.0 (3)
N1—Cd1—O1—C9	14.9 (3)	C4—C5—C6—C1	−0.2 (4)
O3—Cd1—O1—C9	96.11 (17)	C4—C5—C6—N2	−179.0 (3)
Cl1—Cd1—O1—C9	−174.53 (16)	C1—N1—C7—N2	−0.2 (3)
O2—Cd1—O1—C9	2.38 (15)	Cd1—N1—C7—N2	−176.73 (14)
Cl1^i^—Cd1—O1—C9	−84.32 (16)	C1—N1—C7—C8	−177.4 (2)
N1—Cd1—O2—C9	−176.28 (15)	Cd1—N1—C7—C8	6.1 (3)
O1—Cd1—O2—C9	−2.39 (15)	C6—N2—C7—N1	0.1 (3)
O3—Cd1—O2—C9	−89.88 (16)	C6—N2—C7—C8	177.4 (2)
Cl1—Cd1—O2—C9	3.6 (2)	N1—C7—C8—C8^ii^	94.0 (3)
Cl1^i^—Cd1—O2—C9	91.64 (16)	N2—C7—C8—C8^ii^	−82.9 (3)
N1—Cd1—O3—C11	64.7 (3)	Cd1—O2—C9—O1	3.9 (2)
O1—Cd1—O3—C11	−86.7 (3)	Cd1—O2—C9—C10	−174.6 (3)
Cl1—Cd1—O3—C11	176.5 (3)	Cd1—O1—C9—O2	−4.7 (3)
O2—Cd1—O3—C11	−34.5 (3)	Cd1—O1—C9—C10	173.9 (2)

Symmetry codes: (i) −*x*+1, −*y*+2, −*z*+2; (ii) −*x*+1, −*y*+2, −*z*+1.

### Hydrogen-bond geometry (Å, °)

**Table:**

*D*—H···*A*	*D*—H	H···*A*	*D*···*A*	*D*—H···*A*
N2—H2A···O2^ii^	0.86	2.04	2.791 (3)	145
O3—H3B···O1^iii^	0.82	1.83	2.646 (3)	175

Symmetry codes: (ii) −*x*+1, −*y*+2, −*z*+1; (iii) −*x*+2, −*y*+2, −*z*+2.
